# Electric-Field Control of Photon Indistinguishability in Cascaded Decays in Quantum Dots

**DOI:** 10.1021/acs.nanolett.5c01354

**Published:** 2025-04-18

**Authors:** Gabriel Undeutsch, Maximilian Aigner, Ailton J Garcia, Johannes Reindl, Melina Peter, Simon Mader, Christian Weidinger, Saimon F. Covre da Silva, Santanu Manna, Eva Schöll, Armando Rastelli

**Affiliations:** † Institute of Semiconductor and Solid State Physics, Johannes Kepler University, 4040 Linz, Austria; ‡ Instituto de Física Gleb Wataghin, Universidade Estadual de Campinas, 13083-970 Campinas, Brazil; ¶ Indian Institute of Technology Delhi, 110016 New Delhi, India; § Institute for Integrated Circuits and Quantum Computing, Johannes Kepler University, 4040 Linz, Austria

**Keywords:** Semiconductor quantum dots, Lifetime tuning, Photon indistinguishability, Cascaded decay, p-i-n diode, Quantum confined Stark effect

## Abstract

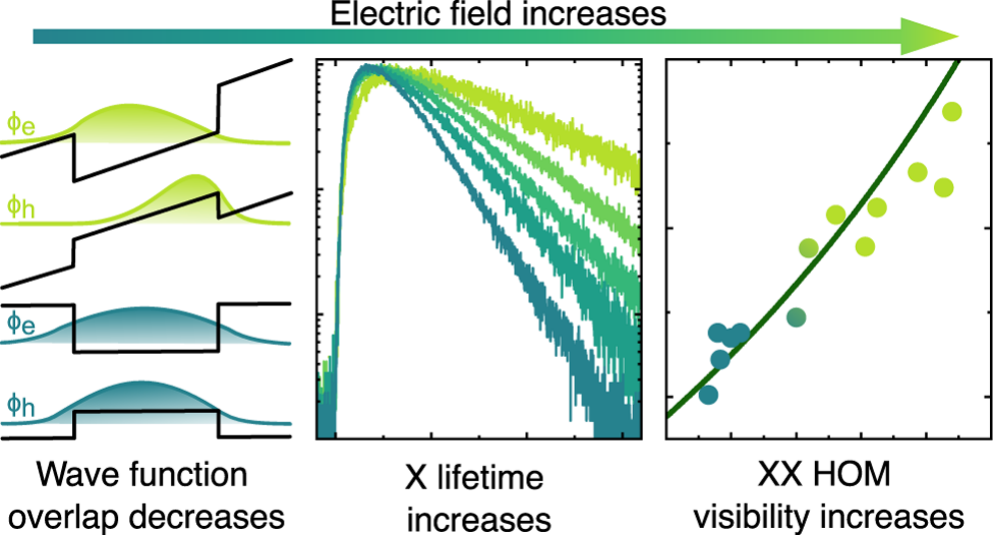

Photon indistinguishability, entanglement, and antibunching are key ingredients in quantum optics and photonics. Decay cascades in quantum emitters offer a simple method to create entangled-photon-pairs with negligible multipair generation probability. However, the degree of indistinguishability of the photons emitted in a cascade is intrinsically limited by the lifetime ratio of the involved transitions. Here we show that, for the biexciton–exciton cascade in a quantum dot, this ratio can be widely tuned by an applied electric field. Hong-Ou-Mandel interference measurements of two subsequently emitted biexciton photons show that their indistinguishability increases with increasing field, following the theoretically predicted behavior. At the same time, the emission line width stays close to the transform-limit, favoring applications relying on the interference among photons emitted by different sources.

In the realm of quantum technologies, many applications require specialized quantum light sources that meet stringent criteria. Among the most sought-after properties is the capability of “on demand” generation of simultaneously highly indistinguishable and strongly entangled photon pairs.^1−3^ Epitaxial semiconductor quantum dots (QDs) have emerged as promising candidates for generating photons with high brightness,^4−6^ high single-photon purity,^7^ narrow line width,^8−11^ and near-unity indistinguishability.^5,12−15^ Additionally, the biexciton (XX) - exciton (X) radiative cascade allows the direct generation of on-demand polarization-entangled photon pairs with near-unity time-averaged fidelities.^16−21^ However, the cascade nature of the process to create entangled photon pairs causes an unwanted temporal entanglement between the two photons, resulting in a nonseparable two-photon state. This reduces the state purity , describing the indistinguishability of the emitted single photons in the time-domain, to^22−24^
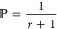
1with the ratio of the radiative lifetimes *r* = τ_*XX*_/τ_*X*_. The purity is experimentally not directly accessible, but – for systems with negligible multiphoton probability, as is the case here – it is identical to the two-photon interference visibility,^25^ which can be measured in Hong-Ou-Mandel (HOM) type experiments. The observed QD lifetime ratio is typically *r* ≈ 0.4 – 0.7,^9,16,24,26^ resulting in a maximum achievable HOM interference visibility of 0.67. It has been proposed and demonstrated that a suitable optical cavity can selectively shorten the XX state (|*XX*⟩) lifetime while keeping the X state (|*X*⟩) lifetime constant, and thereby decrease the lifetime ratio.^23^ However, no increase in photon indistinguishability has yet been shown. In this work, we take a different approach and demonstrate that the lifetime ratio *r* can be conveniently modified by applying a vertical electric field to QDs embedded in a p-i-n diode. The diode structure allows for the charge control of the QD and its environment, enabling stabilization and tuning of the emission properties.^8,10,15,27−31^ In particular, it has been demonstrated that an electric field induces a nonmonotonic variation in the |*X*⟩ lifetime,^32,33^ but we are not aware of similar measurements for the |*XX*⟩.

We focus on GaAs QDs obtained by local droplet etching epitaxy,^9,34^ as these QDs have recently outperformed other systems in terms of degree of polarization-entanglement,^18,31,35^ single photon purity,^7^ photon indistinguishability,^10,14,15^ and spin properties.^36^ We find that an increasing electric field leads to a monotonically increasing |*X*⟩ lifetime, while the |*XX*⟩ lifetime remains almost unchanged. This allows us for the first time to tune the lifetime ratio by an externally applied electric field. With that, we decrease the ratio from 0.64(2) to 0.25(2) and thus increase the theoretically maximum indistinguishability from 0.61(1) to 0.794(8), which is significantly higher than the value typically achieved in nondiode structures. We experimentally verify this by measuring the HOM visibility for XX and X photons. At high fields, the HOM visibility for X photons degrades, most likely due to residual charge noise in the sample, as indicated by line width broadening. However, the XX visibility closely follows the theoretical prediction, with its line width remaining close to the transform-limit even at high fields.

The GaAs QDs used in this work are embedded into a p-i-n diode structure (see Figure 1a and b) and a weak planar cavity built up of distributed Bragg reflectors, with ten pairs below and four pairs above the QD layer (see supplementary). When an external voltage *V* is applied to the diode structure, an electric field  is generated, where *V*_*b*_ is the built-in voltage and *D* is the thickness of the intrinsic layer.^33^ (Note that the n-doped layer is grounded and *V* is the voltage applied to the top p-doped layer.) In our case, we expect *V*_*b*_ to be about +1.7 V as the Al_15_Ga_85_As band gap energy at low temperatures is about 1.73 eV. A higher electric field (lower voltage) leads to a stronger bending of the conduction (CB) and valence band (VB) edges, as seen from the comparison between the calculation results shown in Figure 1a and b for *V* = 0.9 V and *V* = – 2.2 V, respectively. Since in the latter case the energy *E*_*F*_ of the CB quasi-Fermi level lies below the CB edge in the QD region, the QD is in a neutral state and theoretically only neutral excitonic states can be excited.

**Figure 1:**
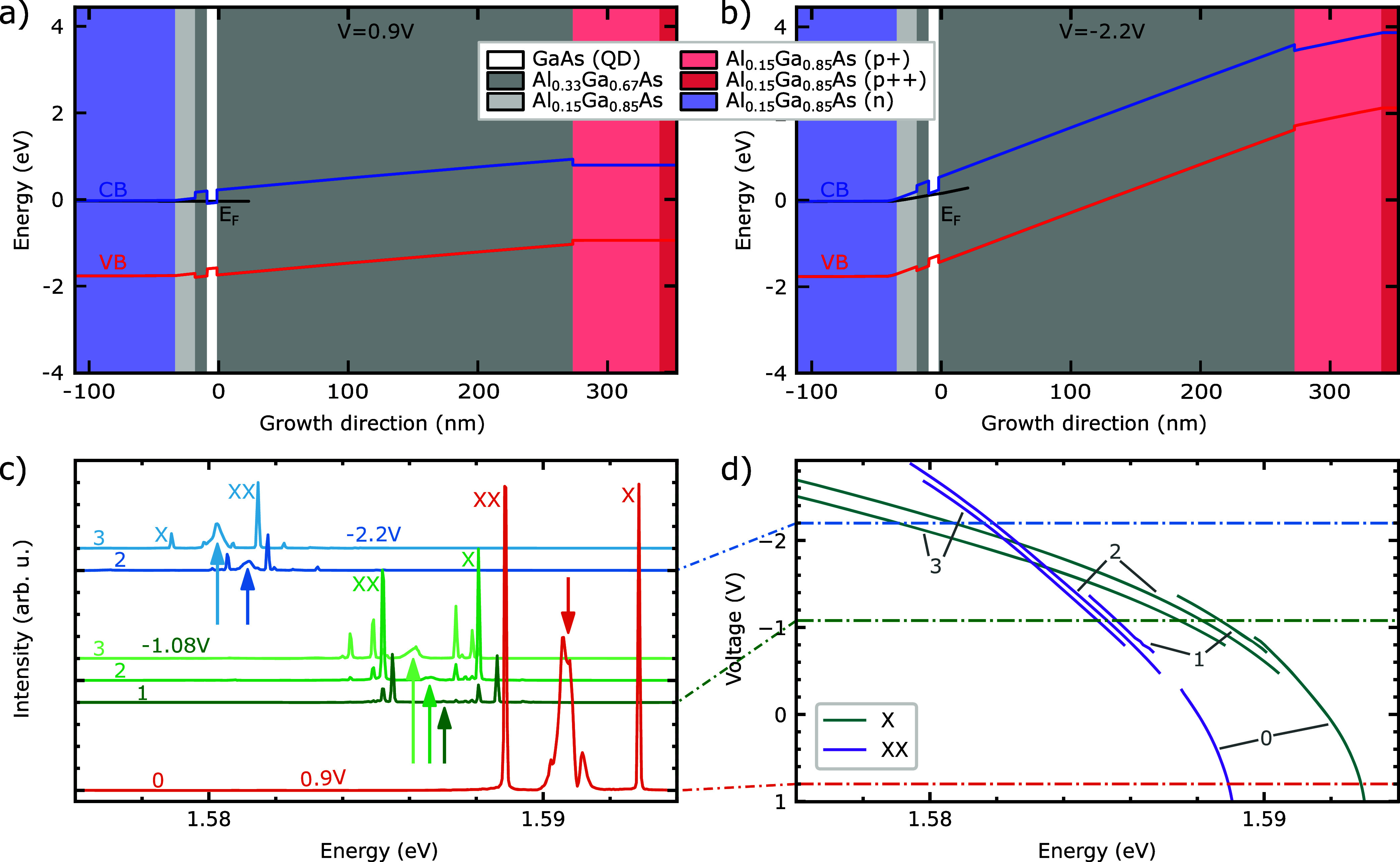
Sample structure with simulated conduction band (CB) and valence band (VB) edges and CB quasi Fermi level (E_*F*_) for (a) an applied voltage *V* = 0.9 V and (b) *V* = −2.2 V. (c) Spectra at three different voltages under π-pulse two-photon excitation (TPE). Depending on the voltage, several biexciton–exciton (XX-X) replicas (labeled 0–3) could be observed, sometimes simultaneously. This is most likely due to different numbers of holes trapped in the vicinity of the QD. For *V* = 0.9 V (orange) only replica 0 is visible. For *V* = −1.08 V (green) and *V* = −2.2 V (blue), three replicas (1–3) and two replicas (2 and 3) are visible. For each spectrum, the laser energy (indicated with arrows) was adjusted to match the TPE resonance. Small contributions from other replicas come from phonon-assisted excitation. (d) Fitted energy of the XX and X photons as a function of voltage. Each data point is extracted from a spectrum under TPE of the respective replica.

To characterize the sample, we perform voltage-dependent photoluminescence measurements of a single QD (QD 1) under resonant excitation of |*XX*⟩ via two-photon excitation (TPE). Representative spectra, collected at different voltages and excitation energies are shown in Figure 1c. For positive voltages, we observe the typical spectrum of GaAs QDs (see example in orange for *V* = +0.9 V), with the dominant XX and X lines stemming from the radiative cascade. At negative voltages, we find several XX and X replicas with slightly different emission energies, see green and blue spectra in Figure 1c. We attribute such replicas to variations in the electric field,^37^ caused by different numbers of holes caught at the tunnel barrier interface close to the QD layer (see supplementary). We study the three most prominent XX-X replicas (labeled as 1–3 according to their energy), which we address by tuning the laser energy to resonantly excite the respective |*XX*⟩. Additional small lines come from other cascades due to phonon-assisted TPE. Figure 1d shows the fitted emission energies for the XX and X photons for varying voltage (similar data for another QD is shown in the supplementary). In a first approximation, the field dependence of the emission energy can be described by a quadratic behavior,^33^ similar to the potential energy of a polarizable electric dipole in an external electric field (see supplementary). Most importantly, we see that the X line red-shifts faster than the XX line with increasing electric field (decreasing voltage). This observation, which is consistent with previous results on InGaAs QDs,^28,38^ indicates that the |*X*⟩ can be more easily polarized than the |*XX*⟩. Intuitively, we attribute this observation to the larger number of charge carriers present in the biexciton complex, partly screening the external field. As a result, for sufficiently large negative voltages, the XX and X emission lines cross and swap their order, as illustrated by the spectra of replicas 2 and 3 at *V* = – 2.2 V in Figure 1c.

In addition to charge and energy tuning, the electric field influences the overlap of the electron and hole wave functions ⟨Φ_*e*_|Φ_*h*_⟩. From a quadratic fit of the X energy we find that, at an applied voltage of *V* ≈ + 1.1 V, the permanent dipole^33,39−42^ present at zero field cancels with the induced dipole, leading to a near maximum achievable wave function overlap, as shown by the schematic in Figure 2a. (Note that in the studied device we cannot reach this point, since for *V* ≳1 V X and XX luminescence is quenched due to single electron charging.) Any change in voltage from this point will pull the wave functions in opposite directions, resulting in a reduced overlap, as sketched in Figure 2b. In a single particle picture, we expect the decay rate to be proportional to the overlap integral of the electron and hole wave functions.^43^ The change in overlap in response to a change in electric field, in turn, depends on the polarizability of the excitonic species. From the observation that the XX line shifts less than the X line for increasing electric field, we can already anticipate that the |*X*⟩ lifetime will increase more than the |*XX*⟩ lifetime with increasing field. This expectation is confirmed by measuring the dynamics of the XX and X emission following TPE, as shown in Figures 2c. Here and in the following measurements, we always use the brightest replica at a given voltage (see supplementary). Figure 2d shows the lifetimes extracted from a fit of the data (see supplementary) as a function of the applied voltage, as well as the resulting lifetime ratio *r*. For positive voltages, both lifetimes stay almost constant with τ_*XX*_ ≈ 110 ps and τ_*X*_ ≈ 175 ps. For negative voltages, the |*XX*⟩ lifetime increases by a factor of 1.5 to 161(4) ps, while the lifetime of the |*X*⟩ increases significantly by a factor of 3.5 to 619(27) ps at *V* = – 2.04 V. Consequently, the lifetime ratio decreases from 0.64(2) to 0.26(1), as shown in orange in Figure 2d. Thus, from eq 1, the theoretical limit for the indistinguishability increases.

**Figure 2:**
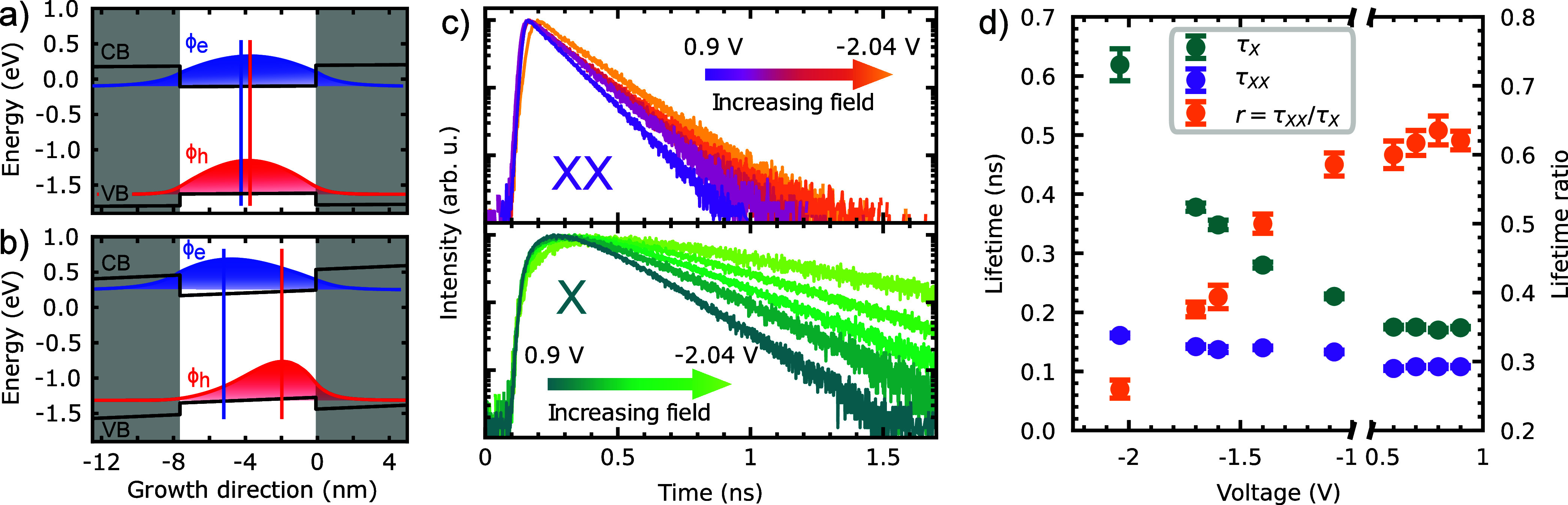
Schematic of electron ϕ_*e*_ and hole ϕ_*h*_ wave functions, showing (a) a high overlap under small electric field vs (b) a reduced overlap under high electric field. (c) Lifetime measurements of the |*XX*⟩ and |*X*⟩ for increasing electric fields (indicated by an arrow) in the specified voltage range. (d) Fitted lifetimes τ as a function of the gate voltage. For decreasing voltage, the relative increase of τ_*XX*_ is much lower than that of τ_*X*_, resulting in a decrease of the lifetime ratio.

To experimentally investigate whether predictions are correct, we measure the two-photon interference visibility for two sequentially emitted XX or X photons in a HOM type interferometer (see supplementary for measurement and analysis details) with a time delay matching the repetition rate of the excitation laser and for different applied voltages. To benchmark the setup and the QDs, we measure the HOM visibility of the resonantly excited negative trion from the same QD at a gate voltage of *V* = +1.03 V, since this is not intrinsically limited by a cascaded emission. From such a measurement, a raw visibility of 0.944(4) and a corrected visibility of 0.991(6) are obtained (see supplementary). Representative photon coincidence histograms for the XX line at *V* = +0.9 V and – 2.04 V are shown in Figure 3a with a horizontal shift of 2 ns for better readability. A decreased central peak is clearly visible for *V* = – 2.04 V in the inset, indicating an improved HOM visibility. The evaluated HOM visibilities for different voltages are shown in Figure 3b and d for the XX and in Figure 3c for the X together with the theoretical limit for the indistinguishability from eq 1. For positive voltages, both the XX and the X show HOM visibilities of ≈0.6, consistent with the constant lifetime ratio. For negative voltages, the XX follows the expected trend and almost reaches the theoretical limit. The highest measured raw (corrected) HOM visibility is 0.735(12) (0.769(13)) at *V* = – 2.04 V (*r* = 0.26), which is close to the theoretical limit of 0.794(8). Figure 3d shows the corrected HOM visibility of the XX against the lifetime ratio. Additionally, it also includes data from a second QD, further supporting our observations. In contrast to the results obtained for the XX and to the theoretical expectations, the raw (corrected) HOM visibility for the X photons degrades to 0.404(18) (0.420(19)) with decreasing lifetime ratio.

**Figure 3:**
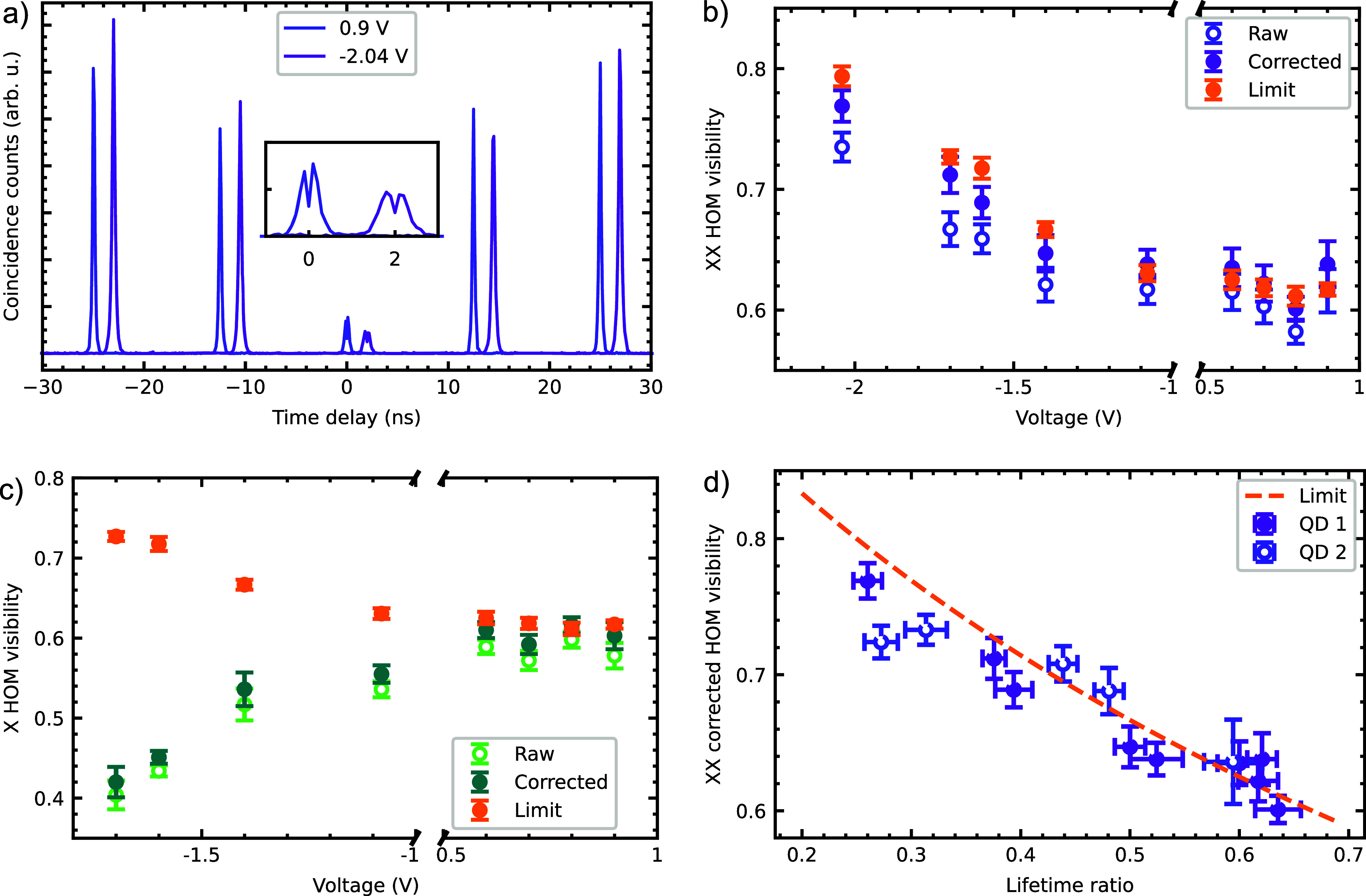
(a) Histograms of Hong-Ou-Mandel (HOM) measurements of the XX photons for the two indicated voltages, with one shifted by 2 ns for better readability. Raw and corrected HOM visibilities for (b) the XX and (c) the X for different applied voltages, as well as the theoretical limit given by eq 1. Whereas the XX follows the expected trend, the X HOM visibility decreases with voltage. (d) Corrected HOM visibility of the XX as a function of lifetime ratio *r* = τ_*XX*_/τ_*X*_. In addition to QD 1, used for all measurements in the Letter, we also show data obtained from another QD (QD 2).

To understand the origin of the indistinguishability drop for X photons and provide a more stringent measurement of the optical quality of the QD emitter at different electric fields, we measure the coherence time of the XX and X lines using a Michelson interferometer (see supplementary). In absence of noise, the upper limit of the coherence time for an X photon is given by τ_*c*_ = 2τ_*X*_, leading to a transform-limited line width of . For the XX, we expect instead .^44^Figure 4a,b shows the first-order correlation function *g*^(1)^(*t*) for the XX and the X respectively for two voltages. At a gate voltage of *V* = +0.9 V, the transform-limited line widths for the XX (X) are 9.88(12) μeV (3.78(4) μeV). The line widths from the Michelson measurements are 11.0(17) μeV (5.4(4) μeV). Therefore, the XX (X) transition is only a factor Γ/Γ_0_ = 1.1(2)(1.4(1)) away from the transform-limit. At a gate voltage of *V* = −1.7 V, the measured line width of the XX is 7.0(11) μeV. Together with the transform-limited line width Γ_0_ = 6.38(10) μeV, this yields the same factor of 1.1(2) as for positive voltages. For the X, the measured line width increases to 8.5(5) μeV, whereas Γ_0_ decreases to 1.74(3) μeV. The X line width is therefore a factor 4.9(3) away from the transform-limit. Figure 4c shows the ratio Γ/Γ_0_ for different gate voltages. It is interesting to note that the XX line width stays close to the transform-limit over the whole voltage range, suggesting that photon indistinguishability is preserved over time separations extending to several minutes (the typical duration of a Michelson interferometry measurement). In contrast, the X line width broadens significantly for decreasing voltages, in line with the drop in HOM indistinguishability shown in Figure 3b. We attribute these observations to residual charge noise and the higher sensitivity of the X transition energy to noise because of its higher polarizability compared to the XX transition energy.^26^

**Figure 4:**
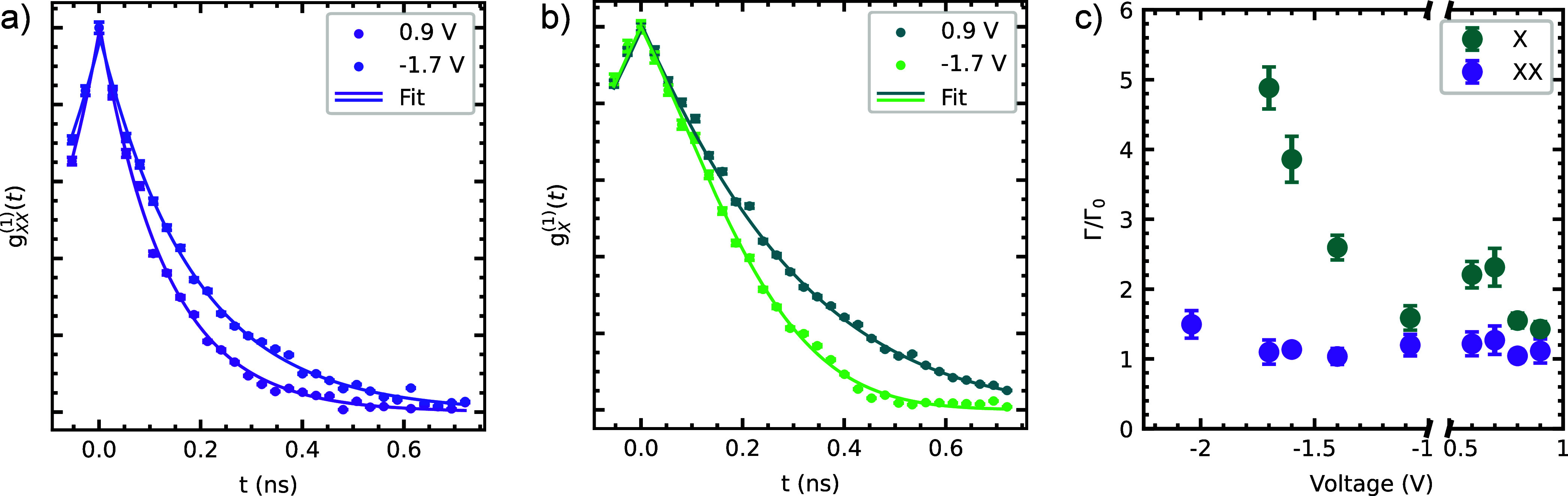
First-order correlation function *g*^(1)^(*t*) as a function of the relative delay *t* of (a) XX and (b) X for two different applied voltages, recorded using a Michelson interferometer. (c) Fitted line widths relative to the respective Fourier transform limit as a function of voltage, showing almost no line broadening for the XX but significant broadening for the X.

In summary, we have demonstrated that the lifetime of transitions in the decay cascade of QDs can be differentially tuned using an external electric field. This reduces the intrinsic limitations on indistinguishability, as confirmed experimentally for the XX photons emitted by GaAs QDs. By operating a p-i-n diode with embedded QDs at negative voltages, strong band bending reduces the overlap of electron and hole wave functions, leading to an increased excited state lifetime. This effect is more pronounced for the |*X*⟩ compared to the |*XX*⟩, due to the higher sensitivity of the exciton to electric field changes compared to the biexciton complex. Our measurements show a reduction in the lifetime ratio from 0.64(2) to 0.26(1). This results in an improved (corrected) HOM interference visibility of 0.769(13) for the XX, approaching the theoretical limit of 0.794(8) – well beyond the values achievable in absence of an electric field. However, the X HOM visibility decreases as the lifetime ratio decreases, which we attribute to an increased sensitivity to noise. Achieving a degree of indistinguishability well above 0.9 remains essential for quantum technology applications. The tuning range of the lifetime ratio could be further increased by dedicated design of the diode structure. Additionally, combining a diode structure with a tailored microcavity, can selectively shorten the |*XX*⟩ lifetime through Purcell enhancement while maintaining the |*X*⟩ lifetime relatively unchanged. These findings, which we expect to apply also to other material systems, may contribute to obtain a quantum light source that simultaneously combines the emission of highly indistinguishable photons with low multiphoton probability, transform-limited line widths and high degree of polarization-entanglement—all key requirements for advancing quantum networks and other quantum technology applications that have long been anticipated.

## Supporting Information Available

Sample: sample structure, band structure simulations; Methods: cryogenic microphotoluminescence setup, autocorrelation measurements, lifetime measurements under TPE and resonant excitation, Hong-Ou-Mandel measurements, Michelson interferometry; Quantum confined Stark effect: polarizability, wave function overlap, permanent dipole; Replicas of the XX-X pairs: origin of the replicas, photogeneration of the holes near QDs, localization of the holes close to the QD, replica intensities, comparison of replicas at the same voltage; Additional Data: indistinguishability of the resonantly excited negative trion, single-photon purity, data on additional quantum dots (PDF)
