# Supplementary material for: Electric-Field Control of Photon Indistinguishability in Cascaded Decays in Quantum Dots
Source: Nano Lett. 2025 Apr 18;25(17):7121–7. doi: 10.1021/acs.nanolett.5c01354 (PMC12046593; doi:10.1021/acs.nanolett.5c01354)
Supplement: Supplementary file 1 — nl5c01354_si_001.pdf [file nl5c01354_si_001.pdf]

Supporting Information: Electric-field control of photon indistinguishability in cascaded decays in quantum dots

Gabriel Undeutsch,[*,†] Maximilian Aigner,[†] Ailton J Garcia Jr,[†] Johannes Reindl,[†] Melina Peter,[†] Simon Mader,[†] Christian Weidinger,[†] Saimon F. Covre da Silva,[†,‡] Santanu Manna,[†,¶] Eva Schöll,[†,§] and Armando Rastelli[*,†]

†*Institute of Semiconductor and Solid State Physics, Johannes Kepler University, Linz, Austria*

‡*Instituto de Física Gleb Wataghin, Universidade Estadual de Campinas, Campinas, Brazil*

¶*Indian Institute of Technology Delhi, India*

§*Institute for Integrated Circuits and Quantum Computing, Johannes Kepler University, Linz, Austria*

E-mail: gabriel.undeutsch@jku.at; armando.rastelli@jku.at

1 Sample

1.1 Sample structure

The GaAs quantum dots (QDs) studied in this paper were fabricated using molecular beam epitaxy with local droplet etching (LDE). The schematic sample structure is shown in Fig. S1. First, a semi-insulating GaAs (001) substrate undergoes in-situ deoxidation, followed by the deposition of a GaAs buffer layer and the growth of a superlattice (30× (2.5 nm

AlAs/2.5 nm GaAs)) to planarize the surface and bury any surface impurities. A second GaAs buffer layer and a planar distributed Bragg reflector (DBR) composed of 10 pairs of $Al_{0.95}Ga_{0.05}As/Al_{0.15}Ga_{0.85}As$ are then grown. An additional $Al_{0.95}Ga_{0.05}As$ layer is incorporated to match the QD position with the electric field antinode of the planar $5\lambda/2$ cavity, which is formed by four additional DBR pairs to improve the extraction efficiency. The QDs are embedded in a p-i-n diode structure. On the n-side, below the QDs, the diode features a 78 nm thick $Al_{0.15}Ga_{0.85}As$ layer doped with Si ($n_n = 7.4 \cdot 10^{17}$ cm^{-3}), serving as an electron reservoir for the QDs. The lower 15% Al concentration prevents the formation of deep donor levels (DX centers).[S1,S2] A tunneling barrier between the n-doped layer and the QDs is formed by a 5 nm $Al_{0.15}Ga_{0.85}As$ layer grown at low temperature to reduce Si segregation, followed by a 10 nm $Al_{0.15}Ga_{0.85}As$ layer grown at higher temperature, as well as a 15 nm $Al_{0.33}Ga_{0.67}As$ layer. For the QD growth, Al is evaporated onto the $Al_{0.33}Ga_{0.67}As$ layer without an As background, forming droplets that etch approximately 8 nm deep nanoholes in the surface during exposure to a reduced As flux. These nanoholes are subsequently filled with GaAs to form the QDs, which are then capped with an $Al_{0.33}Ga_{0.67}As$ layer. Further details are provided in.[S3] The QDs are positioned about 275 nm below the carbon-doped p+-type layer of the diode, which consists of a 65.6 nm thick $Al_{0.15}Ga_{0.85}As$ layer with low doping concentration ($n_{p+} = 5 \cdot 10^{18}$ cm^{-3}), followed by a 13 nm thick highly doped $Al_{0.15}Ga_{0.85}As$ layer ($n_{p++} = 9 \cdot 10^{18}$ cm^{-3}). The top DBR is capped with 4 nm of GaAs to protect the AlGaAs layers from oxidation. To contact the n-layer, trenches are etched using sulfuric acid, followed by deposition of Ni/AuGe/Ni/Au and an annealing step at 420 °C for two minutes in a forming gas environment. For the p-contacts, Pt/Ti/Pt/Au is used, annealed at 400 °C for two minutes.

1.2 Band structure simulation

The simulation results in this work were obtained with nextnano++, a software for semiconductor nanodevices for the self-consistent solution of the Schrödinger, current, and Poisson

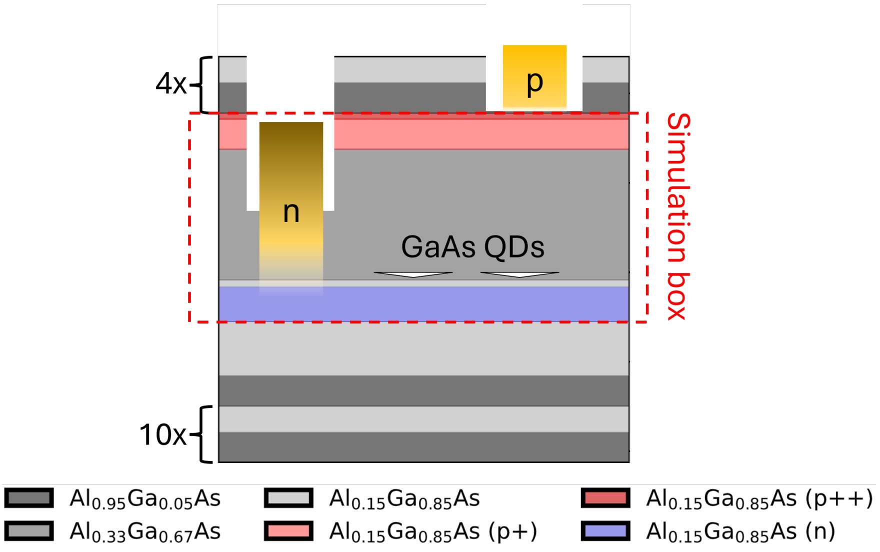

Figure S1: Schematics of the sample structure, including the simulation box.

equation.[S4,S5] For the simulation, the temperature was set to 5 K. Fig. S1 shows an illustration of the contacted diode, including the region over which the simulation was performed (simulation box).

Contacts: The layer thicknesses and doping concentrations defined in the simulation were taken from the growth protocol. For the intrinsic layer, a background carbon-doping of $1 \cdot 10^{15}$ cm^{-3} was assumed to account for the carbon background in the growth chamber. For the donor and acceptor energy, realistic values of 5.8 meV and 27 meV were used.[S6,S7] As a result, the free charge carriers in the structure freeze out when running simulations at low temperatures.

The n- and p-type contacts were defined as Fermi contact, which results in a pinning of the electron and hole quasi Fermi level to the bias applied to the contact. At voltages well below the built-in voltage, the electric field within the intrinsic region is high enough to saturate the electron and hole drift velocity. Therefore, a high field mobility model was used to limit the electron and hole drift velocity to $0.5 \cdot 10^7$ cm/s and, $0.7 \cdot 10^7$ cm/s respectively.[S8,S9]

Energy levels and wave functions: By solving the Schrödinger equation numerically, nextnano++ provides solutions for the energy levels and wave functions within a pre-defined quantum region. At the boundaries of the quantum region, Dirichlet boundary conditions were applied. In the one-dimensional simulation, the GaAs QD was included by a quantum well with a thickness of 7.5 nm. The thickness was chosen such that the calculated transition energy between the lowest electron and heavy hole state roughly matches the measured exciton (X) emission energy.

Observations: The simulated Stark shift of the quantum well is a symmetric function of the electric field, i.e. the emission energy reaches its maximum at zero field, when the applied voltage is equal to the built-in voltage of the diode ($\approx 1.7\,\mathrm{V}$). In reality, we observe an asymmetric Stark shift for the quantum dots in our diode structures, i.e. the maximum emission energy is reached at voltages well below the built-in voltage. We attribute this behavior to the existence of a permanent dipole moment of the charge complexes in the quantum dot.

For negative voltages, the intrinsic region is fully depleted, and the bands are strongly tilted. For positive voltages, the bands become flatter and the charge carrier density in the intrinsic region increases. When the electron density is sufficiently high in the vicinity of the QD, the electron quasi Fermi level is stable in this region and becomes insensitive to small voltage changes. At negative voltages, the electron and heavy hole wave functions are pulled to opposite sides of the quantum well, leading to a reduced overlap.

2 Methods

2.1 Cryogenic micro-photoluminescence setup

Our cryogenic micro photoluminescence spectroscopy setup is shown in Fig. S2 a). The sample is cooled to 4.5 K in a dry closed-cycle cryostat and can be moved in x, y and z

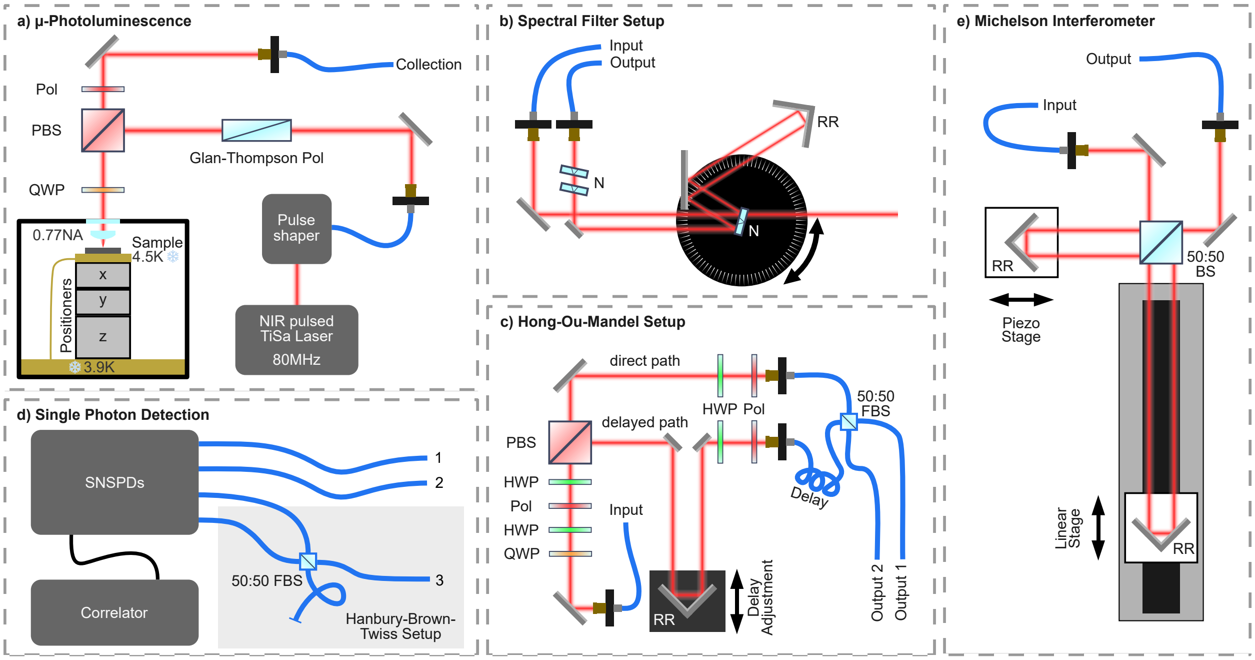

Figure S2: Sketch of the used setup. The parts are separated as indicated and are connected during the measurements using optical fibers. a) cryogenic micro Photoluminescence setup b) spectral filter setup based on volumetric Bragg grating notch filters (N), c) Hong-Ou-Mandel setup to measure the two-photon interference. d) Single photon detection with superconducting nanowire single-photon detectors (SNSPD) and correlator, as well as the Hanbury Brown and Twiss setup to measure the single-photon purity. e) Michelson interferometer to measure the coherence length of the photons. Pol: (linear) polarizer, PBS: polarizing beam splitter, Q(H)WP: quarter (half) wave plate, RR: retro reflector, (F)BS: (fiber) beam splitter

directions using nano positioners. The QD is excited using a pulsed titanium-sapphire laser with a repetition rate of 80 MHz, which is shaped to a pulse length of about 5 ps using a 4f pulse shaper. The laser is sent through a Glan-Thompson polarizer to set a well-defined s-polarized state, which is then reflected at the subsequent polarizing beam splitter cube (PBS). This directs the beam into the cryostat, where it is focused onto the sample using a NA=0.77 cold lens. The same lens is used in our setup with confocal geometry to collect the emission and back reflected laser. The laser is filtered by cross-polarization, and only the p-polarized emission of the sample passes the PBS and an additional linear polarizer (Pol). The detected signal is coupled into a single mode (SM) fiber and can be flexibly sent to different filtering and analysis setups. A quarter wave plate (QWP) between the PBS and cryostat compensates small birefringence effects to improve the laser suppression. The spectra are recorded using a CCD camera connected to a spectrometer (not shown). For all correlation measurements described below, spectral filtering of the single photons is necessary. We use volumetric Bragg grating notch filters (N), which reflect a narrow spectral range of about 200 pm, while transmitting the rest. The central wavelength of reflection can be adjusted by changing the incidence angle and can be tuned by roughly 15 nm. The setup shown in Fig. S2 b) features one N on a rotation stage reflecting the desired wavelength and two to four further N for additional filtering of unwanted spectral components. This filtering setup has a fiber-to-fiber efficiency of $\approx 50\%$.

2.2 Autocorrelation measurements

The measurement of the second-order autocorrelation function $g^{(2)}(t)$ is realized in a Hanbury Brown and Twiss experiment. After spectral filtering, the QD emission is sent into a fiber-coupled 50:50 beam splitter (BS) (input 3 in Fig. S2 d)). The BS outputs are connected to two superconducting nanowire single photon detectors (SNSPDs). The detection events are recorded as time tags by a time tagger, which are then correlated using a PC, to build a histogram of coincidences as a function of time delay between the events. The detectors have

time jitters of 13-14 ps. Together with the time jitter of the time tagger, this results in a total jitter of about 15 ps for single events and about 21 ps for correlations between two detectors. The single-photon purity can be extracted by evaluating the number of coincidences in the central peak at $t = 0$ relative to the average number of coincidences in the 5 side peaks on both sides.

2.3 Lifetime measurements

Lifetime measurements of the exciton and biexciton states ($|X\rangle$, $|XX\rangle$) are performed by time-correlated single photon counting. The filtered QD emission is sent to one SNSPD. A second output of the pulse shaper setup is used to tap off the roughly 5 ps long excitation laser pulses and send it to another SNSPD. The arrival time of the QD photons with respect to the excitation laser pulse is extracted by correlating the time tags of both detectors. The finite rise time of the laser and finite setup timing resolution are taken into account by measuring the instrument response function (IRF). For the XX decay, we assume a monoexponential decay with the $|XX\rangle$ lifetime τ_{XX}. The X decay follows instead a biexponential decay, with the $|XX\rangle$ and $|X\rangle$ lifetimes as time constants. Both formulas are convoluted with the IRF to fit the obtained histograms. An example measurement of QD 4 is shown in Fig. S3 b). To verify the reliability of the $|X\rangle$ lifetime measurements obtained under TPE, we compare it using strictly resonant excitation, as shown in Fig. S3 a). Using this excitation method, the $|X\rangle$ is excited into a superposition of both fine structure components, which leads to a state precession with frequency $\nu = \frac{E_{FSS}}{h}$ and thereby to a time-dependent polarization state of the emitted photon.[S10,S11] Due to the employed cross-polarized excitation/collection configuration, this results in a periodic modulation of the exponential decay, as shown in Fig. S3 a). Note that, under TPE, the $|X\rangle$ is populated by the decay of the $|XX\rangle$ in a superposition state. The $|X\rangle$ state thus precesses with the same frequency quoted above, but the phase differs with each excitation cycle, depending on the polarization of the XX photon. This leads to an effectively unpolarized emission of the X photon and therefore no periodic

modulations in the decay. Determining the lifetimes under these excitation methods thus requires different fitting functions. We extract $\tau_{X,res} = 169(1)$ ps and $\tau_{X,TPE} = 173(3)$ ps, which are in close agreement.

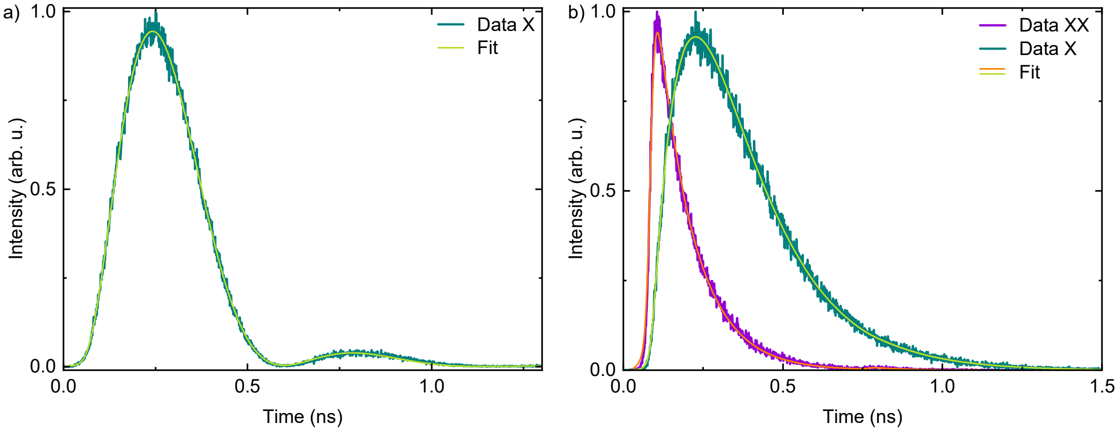

Figure S3: Lifetime measurements for a) the $|X\rangle$ under resonant excitation and b) the $|X\rangle$ and $|XX\rangle$ under TPE. From the fit described above, we extract $\tau_{X,res} = 169(1)$ ps, $\tau_{XX,TPE} = 112(3)$ ps and $\tau_{X,TPE} = 173(3)$ ps.

2.4 Hong-Ou-Mandel measurements

According to the Hong-Ou-Mandel (HOM) effect, if two indistinguishable photons simultaneously impinge on the two different entry ports of a 50:50 beam splitter (BS), they will interfere and will always exit the BS together at the same output port. The measurement of the HOM visibility is therefore a good measure for the photon indistinguishability.

2.4.1 Setup

The HOM interference visibility of subsequent photons from one source is usually measured using an unbalanced Mach-Zehnder-interferometer, as shown in Fig. S2 c). The stream of filtered single photons is sent into the setup using a SM fiber (Input). To correct for the random unitary transformation of the polarization by the fiber, a combination of half-wave plate (HWP) and quarter-wave plate (QWP) is used to maximize the transmission through the following polarizer (Pol). The HWP in front of the PBS is set to send 50% of the intensity

in each interferometer arm. The arms have different lengths to introduce a delay of 12.5 ns in one arm, which matches the 80 MHz repetition rate of the excitation laser. This delay is achieved by a combination of a 2 m-long fiber and an adjustable free-space delay. Each path contains a HWP and Pol before a polarization maintaining (PM) fiber connected to a fiber-coupled 50:50 BS, where the interference will take place. The Pols set the polarization of the photons entering the BS and are set to the same (perpendicular) polarization for co (cross)-polarized measurements. Furthermore, they are aligned along one polarization axis of the fiber. The HWPs compensate for the different efficiencies in the two arms and are adjusted such that the total transmitted intensity from each arm is equal. The two exits (Output 1 and Output 2) are connected to two SNSPDs (1 and 2 in Fig. S2 d)) and correlated as described in Sec. 2.2.

2.4.2 Measurements and data evaluation

To determine the HOM visibility, correlation measurements with co- and cross-polarization have to be carried out. In the case of the co-polarized measurement, the photons are ideally indistinguishable, interfere, and thus always leave the BS together. This leads to no coincidences at zero time delay after correlation of the two outputs. For the cross-polarized measurement, the photons are fully distinguishable, and therefore leave the BS at opposite outputs with a probability of 50%. The expected relative intensities of the central histogram peaks are therefore ... 1, 1, 0.75, 0(0.5), 0.75, 1, 1 ... for fully indistinguishable (distinguishable) photons.

To analyze the data, first, the center peak area $A_{\text{center, } j}$ for each measurement ($j \in \{\text{co, cross}\}$) is normalized with the average side peak area for absolute delays $\geq 2 \cdot 12.5$ ns: $a_j = \frac{A_{\text{center, } j}}{A_{\text{side, } j}}$. The raw HOM visibility can then be determined by

$$V_{\text{HOM,raw}} = 1 - \frac{a_{\text{co}}}{a_{\text{cross}}}. \quad (1)$$

The analysis described here is only valid for a perfect interference BS with splitting ratio $R : T = 50 : 50$, perfect classical interference visibility $\nu = 1$, and perfect single photons ($g^{(2)}(0) = 0$). For deviations, the obtained raw HOM visibility can be corrected by:[S12,S13]

$$V_{\mathrm{HOM,corr}} = V_{\mathrm{HOM,raw}} \frac{1 + 2g^{(2)}(0)}{\nu^2}. \quad (2)$$

By sending a narrow cw laser through our setup we measured a classical visibility of $\nu = 0.985$. The multi-photon emission probability values for each measurement are listed below in Sec 5.2.

2.5 Michelson interferometry

Michelson interferometry, i.e., the measurement of the first-order coherence $g^{(1)}(t)$, is used to determine the coherence properties of a light source and, thereby, further quantify its optical quality. A sketch of a Michelson interferometer is given in Fig. S2e). The incoming signal is evenly divided into two interferometer arms using a 50:50 beam splitter cube (BS). Each arm contains a retroreflector (RR) mounted on a linear motorized stage (travel range 30 cm) and a linear piezo stage (travel range 2 mm) respectively, to redirect the signal back to the BS, where the two beams interfere. One output of the BS is coupled into a multimode fiber and sent to the spectrometer to record the intensity. The integrated intensity of the selected emission line is recorded as a function of the piezo stage position in steps of 20 nm. This changes the relative path length, causing a phase shift that generates an interference pattern. The resulting sinusoidal intensity profile is fitted with a cosine function. This allows us to extract the interference fringe visibility by

$$v = \frac{I_{max} - I_{min}}{I_{max} + I_{min}}. \quad (3)$$

This measurement is repeated for increasing path length of the second arm (i.e. different positions of the linear stage) until the interference fringes disappear and therefore $v \approx 0$.

Here, we assume that the path length difference introduced by the piezo stage is negligible in comparison to the path length difference introduced by the linear stage. The visibility as a function of the relative delay time, i.e. the path length difference divided by the speed of light, is fitted using the Fourier-transform (FT) of a Voigt function, which is the convolution of a Gaussian and a Lorentzian. From the fit we extract an approximate linewidth[S14] $\Gamma \approx 0.5346 f_L + \sqrt{0.2166 f_L^2 + f_G^2}$, with the FWHM widths in energy domain $f_{L,G}$ of the Lorentzian and Gaussian contribution.

3 Quantum confined Stark effect

Figure S4 a) shows a 2D plot with spectra of several replicas of QD 1 under two-photon excitation at different voltages. The pump laser energy was tuned to the resonance of each replica pair. In order to present the data more clearly, only the replicas are shown, whereas residual laser and other small emission lines are cut out. This is done by showing only a few pixels around each emission line for each spectrum. Each emission line is fitted with a Gaussian function to obtain the energy. The results are shown as dashed lines as well as isolated in Fig. S4 b). We fitted these curves using the quadratic equation[S15]

$$E(F) = E_0 - \alpha F - \beta F^2, \tag{4}$$

describing the quantum confined Stark effect. E_0 is the transition energy at zero field, α can be interpreted as the permanent dipole of the $|X\rangle$ and β its polarizability. Here we substituted $F = \frac{V_b - V}{D}$, with the built-in voltage $V_b = +1.7\,\mathrm{V}$ and the thickness of the intrinsic layer $D = 305\,\mathrm{nm}$. Figure S4 c) shows the integrated intensity of the emission lines, corresponding to the XX and X replicas in Fig. S4 b). The XX0 and X0 are most prominent around 0.8 V. Towards lower voltages, the emission is quenched due to charging of the QD. Around −0.8 V, the replicas appear, with XX2 and X2 being the brightest for almost the entire range. The maximum of the summed up intensities of all replicas is similar to the

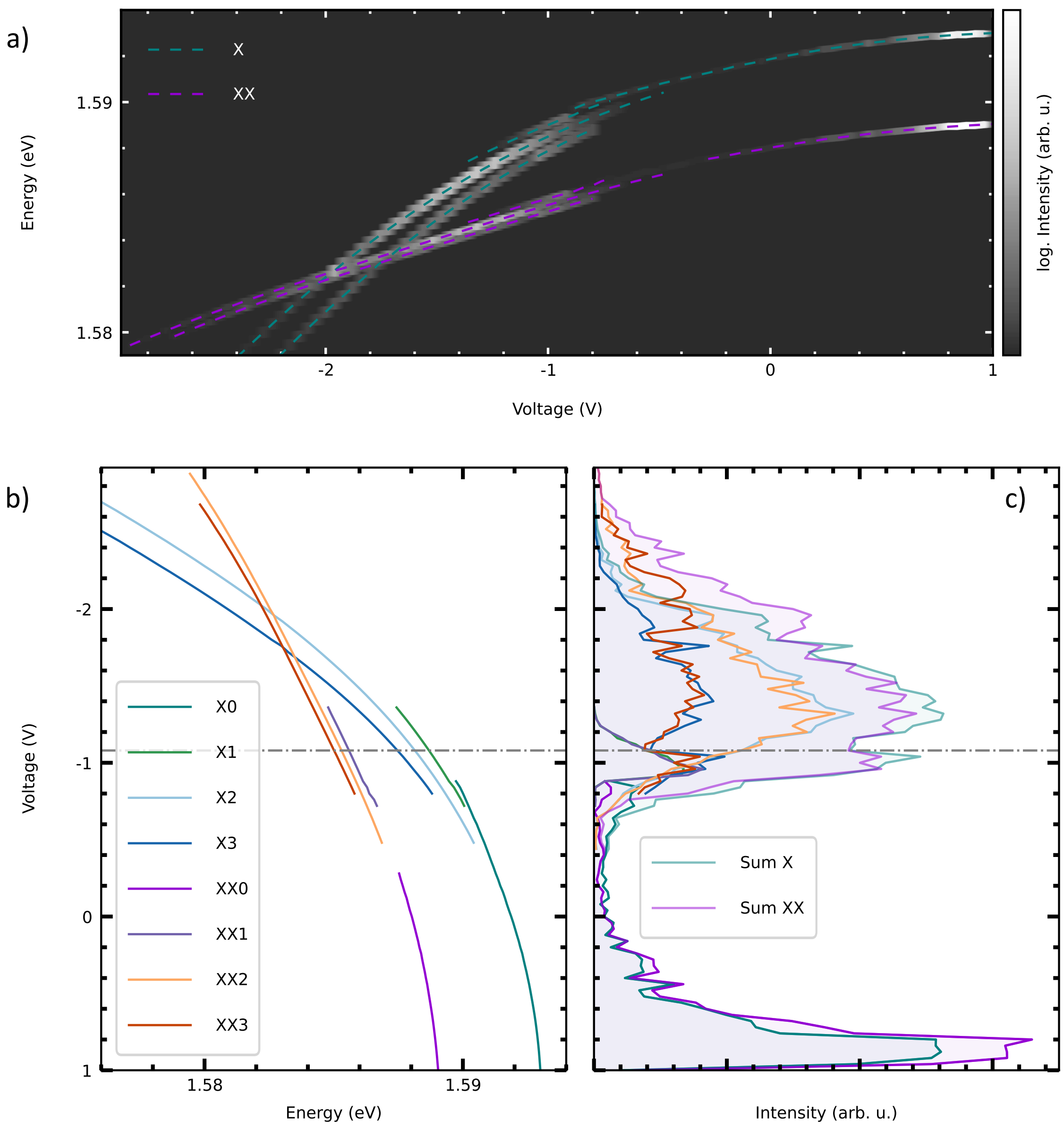

Figure S4: a) Spectra under two-photon excitation of several XX-X replicas of QD 1 as a function of the voltage. See text for the reconstruction of the plot. All emission lines are fitted using Gaussian functions. The fitted energies are plotted as dashed lines and displayed in b). Note that b) corresponds to Fig. 1 c) in the main paper. c) Intensities of the XX and X replicas as well as total XX and X intensity independent of replicas. The gray dash-dotted line indicates −1.08 V, where we compared replicas 1–3 (table S2).

intensity of the XX0 and X0, where the QD is blinking-free. From this, one can argue that the total emission efficiency of all the replicas together is as high as around 0.8 V. At even higher negative voltages, additional replicas appear, which are not studied here.

Table S1: Fit values for XX and X of each replica of QD 1 from Fig. S4

Replica	0	1	2	3
$E_{0,XX}$ (eV)	1.589018(8)	1.5904(6)	1.59217(8)	1.59136(13)
$E_{0,\|XX\rangle}$ (eV)	3.181929(10)	3.1811(7)	3.17913(12)	3.1783(2)
α_{XX} $(\frac{\text{eV}\cdot\text{nm}}{\text{V}})$	-0.142(4)	0.21(12)	0.629(16)	0.53(3)
$\alpha_{\|XX\rangle}$ $(\frac{\text{eV}\cdot\text{nm}}{\text{V}})$	-0.335(6)	-0.61(15)	-1.03(3)	-1.20(4)
β_{XX} $(\frac{\text{eV}\cdot\text{nm}^2}{\text{V}^2})$	58.2(6)	35(7)	13.6(7)	18.0(11)
$\beta_{\|XX\rangle}$ $(\frac{\text{eV}\cdot\text{nm}^2}{\text{V}^2})$	125.2(7)	149(8)	181.5(11)	202.0(19)
$\frac{\beta_{\|XX\rangle}}{2e}$ $(\frac{\text{nm}^2}{\text{V}})$	62.6(4)	75(4)	90.8(5)	101.0(10)
$E_{0,\|X\rangle}$ (eV)	1.592911(7)	1.5906(4)	1.58697(9)	1.58700(15)
$\alpha_{\|X\rangle}$ $(\frac{\text{eV}\cdot\text{nm}}{\text{V}})$	-0.193(4)	-0.83(8)	-1.66(17)	-1.73(3)
$\beta_{\|X\rangle}$ $(\frac{\text{eV}\cdot\text{nm}^2}{\text{V}^2})$	67.0(4)	114(5)	167.9(8)	184.0(15)
$\frac{\beta_{\|X\rangle}}{e}$ $(\frac{\text{nm}^2}{\text{V}})$	67.0(4)	114(5)	167.9(8)	184.0(15)

Table S1 shows the fit parameters of the zero field energy E_0, the permanent dipole α and the polarizability β for XX and XX photons for the replicas 0–3 in the data shown in Fig. S4 b). In addition to the values obtained for the XX and X lines, it is interesting to evaluate also the values of E_0, α and β for the excitonic states giving rise to these emission lines. For the $|X\rangle$, these directly correspond to the fit parameters. For the $|XX\rangle$, the respective fit parameters of both photons have to be summed up: $E_{0,|XX\rangle} = E_{0,XX} + E_{0,X}$, $\alpha_{|XX\rangle} = \alpha_{XX} + \alpha_X$ and $\beta_{|XX\rangle} = \beta_{XX} + \beta_X$. Although we used a quadratic fit function, we expect the real function for $E(F)$ to be slightly asymmetric, as the QD potential is asymmetric in the growth direction.

3.1 Polarizability

Since the $|XX\rangle$ comprises the double number of charges compared to the $|X\rangle$, we introduce $\frac{\beta}{q} \equiv \frac{\partial d}{\partial F}$ to better compare both states. This quantity describes the change of the average distance d of the charges in the dipole under electric field changes. We use this to characterize the change in wave function overlap. For the $|X\rangle$, the charge of the dipole is $q = e$, whereas it is $q = 2e$ for the $|XX\rangle$. As seen in table S1 $\frac{\beta}{q}$ is higher for the $|X\rangle$ than for $|XX\rangle$ for all replicas. Whereas for positive voltages (replica 0), the values are similar, the difference increases with increasing field and $\frac{\beta_{|X\rangle}}{e}$ becomes more than $1.5\times$ higher than $\frac{\beta_{|XX\rangle}}{2e}$. This is visible in Fig. S4 b), as the XX energies show much lower curvatures compared to the X energies.

3.2 Wave function overlap

The lower polarizability of the $|XX\rangle$ can be qualitatively interpreted as a partial screening of the external electric field. Correlation effects, which are more pronounced for the $|XX\rangle$ compared to the $|X\rangle$, will tend to minimize Coulomb repulsion among carriers of the same sign and maximize electron-hole attraction for any given external field, thus reducing the effect of the external perturbation. This leads to a higher sensitivity of the $|X\rangle$ to changes in the electric field. In addition to the higher tunability of the X emission energy, higher fields also lead to a lower wave function overlap compared to the $|XX\rangle$. This results in tunability of the $|X\rangle$ lifetime by a factor of 3.5. Furthermore, the $|X\rangle$ also shows a higher sensitivity to charge noise, leading to increased linewidth broadening. For the $|XX\rangle$, on the other hand, the lifetime increases less and the linewidth remains transform limited.

3.3 Permanent dipole

The shortest $|X\rangle$ lifetime we measured is $\approx 175\,\mathrm{ps}$ at an applied voltage of $V \simeq 1\,\mathrm{V}$. Unfortunately, we cannot access the neutral X in the zero electric field region ($V = V_b \simeq 1.7\,\mathrm{V}$), since

the QD emission is dominated by highly negatively-charged excitonic states in this regime. For comparison, GaAs QDs in non-diode samples exhibit $|X\rangle$ lifetimes of $\approx 200-250\,\mathrm{ps}$.[S3] This suggests that the electron-hole wave function overlap is not maximal in non-diode samples (zero external electric field). Specifically, from the quadratic fit of the data for QDs measured in this work, we find that the permanent electric dipole at zero field is negative (see negative values of α_X in Table S1), indicating that the barycenter of the hole wave function is located below the electron in growth direction. Interestingly, this result is different from that obtained in Ref.,[S16] where a dipole with opposite sign was observed, possibly due to strain-induced effects due to bonding on a different substrate in the QDs studied there or to differences in the structural properties of the QDs. A permanent dipole has previously been observed in several works on InGaAs QDs[S15,S17–S20] and its sign manipulated by strain.[S21] With increasing fields ($V \approx 1-0.5\,\mathrm{V}$), the charge carriers are first pushed closer together (increasing the wave function overlap and thus shortening the lifetime) until they swap positions and the electron is located in the tip of the nanohole. Now, with increasing fields, the charge carriers are pulled apart, leading to decreasing wave function overlap and therefore increasing lifetime.

4 Replicas of the XX-X pairs

4.1 Origin of the replicas

Since we observe discrete replicas in our spectra, we make discrete changes in the electrical field responsible, which we attribute to discrete numbers of charges in the vicinity of the QD. All investigated QDs show red shifted replicas (see Fig. S4 a,b)), indicating that the additional charge carriers enhance the applied field. Consequently, we expect either electrons above the quantum dot (toward the p-contact) or holes below the quantum dot (on the n-side of the diode). Since the band structure lacks a potential structure to trap electrons, we infer that holes are likely trapped in the triangular-shaped potential within the $Al_{0.15}Ga_{0.85}As$

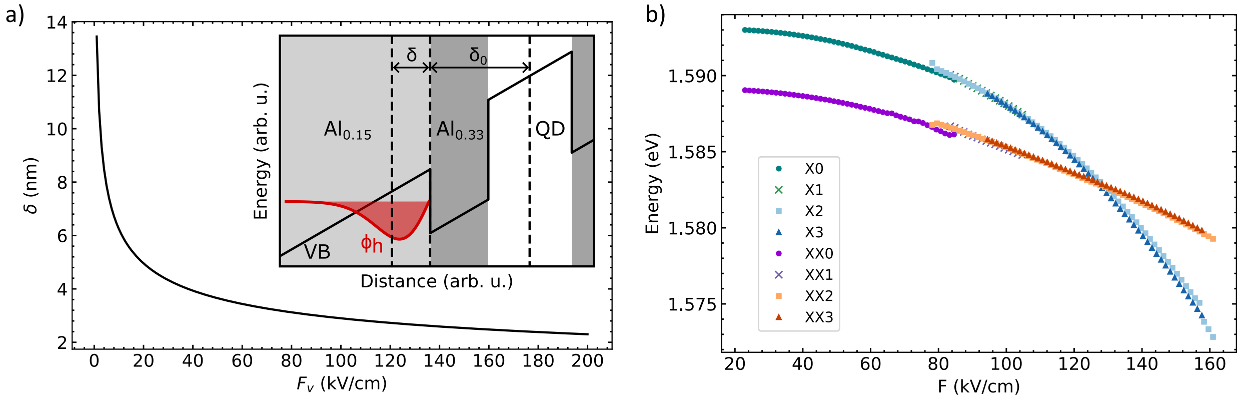

Figure S5: Inset: Zoom in from Fig. 1 b) in the main manuscript: the valence band edge forms a triangular potential below the QD, where holes can be trapped. $\delta_0 = 11.5\,\text{nm}$ is the distance between the $Al_{0.33}Ga_{0.67}As/Al_{0.15}Ga_{0.85}As$ interface and the center of the QD. δ is the field-dependent distance of the trapped hole to the interface. a) Calculated distance δ between the barycenter of the hole wave function in the triangular potential well and the $Al_{0.33}Ga_{0.67}As$ layer, indicated in the inset as a function of the electric field F_v given by the applied voltage. b) Emission energy of the QD 1 replicas as a function of the total electric field F resulting from the applied voltage, and the field created by one, two or three positive charges (and their mirror image at the n-doped layer) at distance $\delta_0 + \delta$.

layer (see Fig. S5 a, inset)).[S22] In order to find out whether these holes could lead to the observed shift in energy, we first examine the situation of a point charge located right below the QD and reduce the situation to a one-dimensional problem. In this scenario, we calculate the field with the contribution of the applied voltage $F_v = \frac{V_b - V}{D}$, the contribution from a point charge F_c at distance $\delta + \delta_0$ from the center of the QD and its mirror charge in the n doped layer F_m. As indicated in the inset of Fig. S5 a), $\delta_0 = 11.5\,\text{nm}$ is the distance between the center of the QD and the interface between the $Al_{0.15}Ga_{0.85}As$ and $Al_{0.33}Ga_{0.67}As$ layers. δ is the distance between the barycenter of the trapped additional hole wave function and the interface. Since the triangular well changes with the applied field, δ is also voltage dependent. This is calculated by solving the Schrödinger equation for a triangular well resulting in Airy functions, as plotted in Fig. S5 a). δ is determined by the expectation value. From this, the resulting electric field F is calculated to be:

$$F = F_v + n \frac{1}{(\delta(F_v) + \delta_0)^2 \cdot 4\pi\epsilon_0\epsilon} + F_m \tag{5}$$

with n being the number of holes (1 for X1/XX1, 2 for X2/XX2, 3 for X3/XX3), ϵ_0 being the vacuum permittivity and ϵ being the permittivity in $Al_{0.33}Ga_{0.67}As$ at 4 K, extrapolated from literature.[S23,S24] Figure S5 b) shows the emission energies of QD 1 as a function of the corrected field F. It shows that the XX and X emission lines of all replicas now nicely follow the same behavior. We thus conclude that the discrete jumps observed in the spectra are consistent with the presence of a number of n holes at the interface between the two AlGaAs layers, right below the QD.

What remains to be explained is how holes are generated and why they seem to get trapped right below the QD rather than at an arbitrary location in the plane of the interface.

4.2 Photogeneration of holes near QDs

As discussed above, we expect holes being confined at the triangular potential due to the band bending in the $Al_{0.15}Ga_{0.85}As$ layer. The explanation of the presence of these holes is not trivial, since (i) the electric field leads to full depletion of holes close to the n-region of the diode; (ii) the laser energy ($E_{\mathrm{ph}} \approx 1.59\,\mathrm{eV}$) is smaller than the band gap ($E_{\mathrm{g}} \approx 1.73\,\mathrm{eV}$), prohibiting direct optical excitation of electron-hole pairs; and (iii) possible tunneling of holes from the QD to the triangular potential is blocked by the large tilt of the bands at high electric fields. Instead, we ascribe the generation of these holes to the absorption of laser photons in the undoped $Al_{0.15}Ga_{0.85}As$ layer via the Franz-Keldysh effect, which describes the appearance of an absorption tail below the bandgap energy at high electric fields.[S25] Specifically, a laser photon can generate an electron-hole pair, as illustrated in Fig. S6. The electron wave function is extended over the n-type region with an exponential tail leaking into the $Al_{0.15}Ga_{0.85}As$ layer and the hole is trapped at the triangular potential well. The finite extension of the $Al_{0.15}Ga_{0.85}As$ barrier (thickness: $d = 15.1\,\mathrm{nm}$) does not allow for the absorption of photons at too low electric fields. Instead, we expect an onset of the Franz-Keldysh, when the electric field F in the diode is high enough so that the potential difference $e \cdot F \cdot d$ across the $Al_{0.15}Ga_{0.85}As$ layer compensates the energy difference $E_{\mathrm{g}} - E_{\mathrm{ph}} \approx 0.14\,\mathrm{eV}$.

In our diode structure, this condition is fulfilled for a voltage of $V \approx -1.16\,\mathrm{V}$. Starting from here, the absorption coefficient of the layer increases with decreasing voltage. Indeed, we see the maximum intensity of the replica lines in the voltage range of about $-1.0\,\mathrm{V}$ to $-1.8\,\mathrm{V}$, as visible in Fig. S4 c). We expect these holes to be long-lived compared to the excitonic lifetimes, since electrons from the n-doped layer are pushed away from the interface by the large electric field.

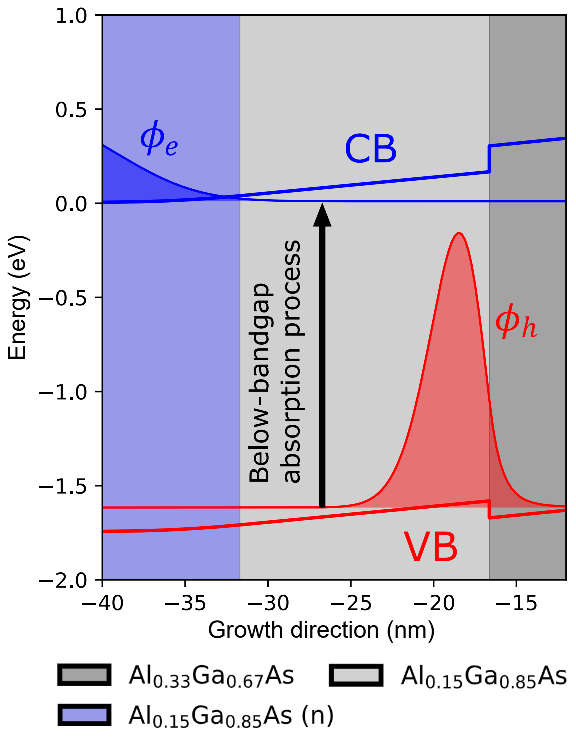

Figure S6: Schematics of the absorption of laser photons via the Franz-Keldysh effect. After absorption, electrons and holes are separated by the electric field with electrons drifting to the n-doped layer and holes getting trapped at the $Al_{0.33}Ga_{0.67}As/Al_{0.15}Ga_{0.85}As$ interface

4.3 Localization of the holes close to the QD

While the confinement of the holes in the triangular well along the growth direction is straightforward, this only serves to trap them in plane. The fact that we observe discrete emissions from the replicas, together with the energy shift observed, leads us to conclude that

the holes are trapped directly below (in growth direction) the QD. This requires a lateral confinement potential, and we explored several possible explanations: (i) the electric dipole of a polarized $|X\rangle$ or $|XX\rangle$ in the QD, producing an attractive potential on holes located at the interface; (ii) the local strain caused by the QD itself due to the small lattice mismatch between GaAs and surrounding barrier; (iii) local interface roughness. Both contributions (i) and (ii) induce an attractive potential well below 1 meV, so that the discrete jumps in emission should disappear at temperatures well above $1\,\text{meV}/k_B \simeq 12\,\text{K}$. To test these ideas, we examined the emission at 30 K to see if the holes would be thermally excited and the replicas would disappear. Since this was not the case, these contributions can not solely explain the lateral localizations of the holes.

Concerning (iii) we note that the rate of formation of Al droplets in the LDE method increases with the roughness of the substrate.[S26] We speculate that an area of increased roughness at the interface between the 15% and 33% Al layers might transfer to the overlying layer for LDE growth and favor the formation of droplets there. This could lead to trapping of holes, specifically directly below the QD.

4.4 Replica intensities

Figure S4 c) shows the integrated intensity of the XX and X replicas separated, as well as the sum of all XX and X emission lines. For almost all voltages, the XX intensity is roughly equal to the X intensity for each replica. From $V = 0\,\text{V}$ to $V = -0.7\,\text{V}$ the overall intensity is very low, since positively charged states are dominant in this region. For lower voltages, replicas 1–3 start to appear, while the initial replica 0 disappears. Replica 2 is predominant for the largest voltage range, namely from $V = -1.08\,\text{V}$ to $-1.7\,\text{V}$. It is therefore used for almost all measurement points in the main paper. Only at $V = -2.04\,\text{V}$ replica 3 is chosen, as it is brighter. After the crossing of the XX and X emission energies, the intensity of the X decreased faster than the XX. This, together with the appearance of even more replicas, limits the maximum tuning range.

4.5 Comparison of replicas at the same voltage

In order to prove that replicas behave similarly, we compare the three replicas (replicas 1-–3) observed at an applied voltage of $-1.08\,\mathrm{V}$, where all replicas show a reasonable brightness. The summarized results in Tab. S2 show that all replicas exhibit similar behavior. We therefore focus on the most prominent replica for each voltage in the main paper, mostly replica 2 (see. Fig. S4 c)). Only at the last data point at $-2.04\,\mathrm{V}$ replica 3 dominated. These findings and the energy shift behavior shown in Fig. S5 makes us confident that replicas are not necessary to tune the ratio between $|XX\rangle$ and $|X\rangle$ lifetime and that a device featuring no trap for holes would still be suitable to this aim.

Table S2: Lifetimes, lifetime ratio r, linewidths Γ, also with respect to their transform limited linewidth Γ_0, $g^{(2)}(0)$, and HOM visibilities of the XX and X for the replicas 1-3, which are present at a voltage of $-1.08\,\mathrm{V}$.

Replica	1	2	3
XX τ (ps)	119(4)	133(3)	122(3)
X τ (ps)	227(7)	227(4)	252(3)
r	0.52(3)	0.59(2)	0.484(13)
XX Γ (μeV)	10(2)	9.4(12)	7.7(2)
XX $\frac{\Gamma}{\Gamma_0}$	1.2(3)	1.2(2)	1.0(4)
X Γ (μeV)	4.9(6)	4.6(5)	5.6(4)
X $\frac{\Gamma}{\Gamma_0}$	1.7(3)	1.6(2)	2.1(2)
XX $g^{(2)}(0)$	0.0035(10)	0.0069(10)	0.0120(5)
X $g^{(2)}(0)$	0.006(6)	0.0070(5)	0.008(1)
raw XX V_{HOM}	0.607(10)	0.617(12)	0.568(14)
corr XX V_{HOM}	0.625(10)	0.638(12)	0.594(15)
raw X V_{HOM}	0.535(12)	0.536(10)	0.501(12)
corr X V_{HOM}	0.553(12)	0.555(12)	0.519(13)

5 Additional Data

5.1 Indistinguishability of the resonantly excited negative trion

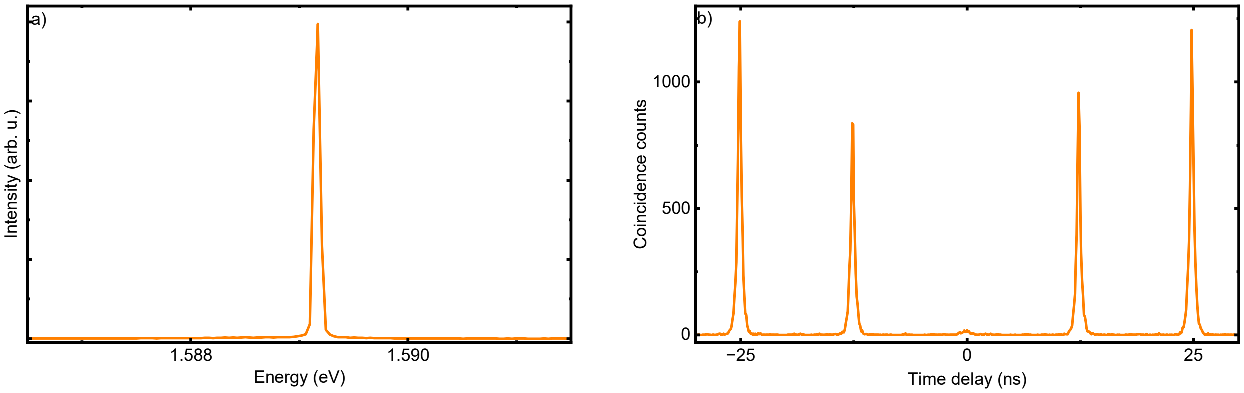

Figure S7: a) Spectrum of the negative trion of QD 1 at a voltage of 1.03 V under resonant fluorescence conditions. b) Histogram of the corresponding HOM measurement.

To assess whether our sample suffers from noise sources or the HOM setup is not well aligned, we measure the HOM interference visibility of photons emitted by a negative trion under resonance fluorescence conditions. The unfiltered spectrum of this transition is shown in Fig. S7 a) and the corresponding coincidence histogram is shown in Fig. S7 b). The measured raw HOM visibility is 0.944(4). Correction for a small multi-photon contribution ($g^{(2)}(0) = 0.009(2)$) and imperfect beam overlap at the beam splitter yield a corrected HOM visibility of 0.991(6).

5.2 Single-photon purity

We measure the second-order autocorrelation function for the XX and X, as described in Sec. 2.2, to verify good filtering of the emission line of interest and for the possibility to correct the measured HOM visibilities for remaining multi-photon contributions. The analyzed $g^{(2)}(0)$ values are listed in Table S3. An example measurement is shown in Fig. S8.

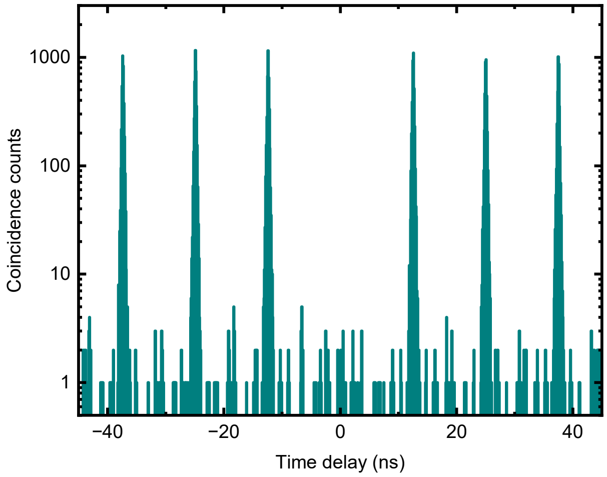

Figure S8: Second-order autocorrelation histogram of the XX at an applied voltage of $-1.08\,\mathrm{V}$ yielding $g^{(2)}(0) = 0.0035(10)$.

Table S3: Single-photon purity $g^{(2)}(0)$ of the XX and X for different voltages.

Voltage (V)	Replica	XX $g^{(2)}(0)$	X $g^{(2)}(0)$
0.9	0	0.008(1)	0.015(2)
0.8	0	0.006(1)	0.004(1)
0.7	0	0.012(6)	0.0070(3)
0.6	0	0.010(7)	0.010(1)
-1.08	1	0.0035(10)	0.006(6)
-1.08	2	0.0069(10)	0.0070(5)
-1.08	3	0.0120(5)	0.008(1)
-1.4	2	0.0151(12)	0.0106(11)
-1.6	2	0.0180(16)	0.0105(6)
-1.7	2	0.039(4)	0.1140(14)
-2.04	3	0.0187(17)	

5.3 Data on additional quantum dots

All other QDs consistently show replicas, however, with different replicas being more or less pronounced. In the following, we present the data of QD 2, included in Fig. 3 d) in the main manuscript, as well as replicas of QD 3.

5.3.1 Data of QD 2

Voltage (V)	0.85	-1.55	-1.8	-2.1	-2.2
Replica	1	3	3	4	4
XX τ (ps)	104(4)	138(3)	154(3)	168(4)	155(5)
X τ (ps)	175(4)	287(5)	351(8)	536(30)	569(26)
r	0.59(4)	0.27(2)	0.31(3)	0.44(2)	0.48(2)
XX $g^{(2)}(0)$	0.007(1)	0.010(1)	0.019(2)	0.006(1)	0.006(1)
X $g^{(2)}(0)$	0.005(1)				
raw XX V_{HOM}	0.608(30)	0.655(17)	0.662(12)	0.701(11)	0.694(12)
corr XX V_{HOM}	0.636(31)	0.688(17)	0.708(13)	0.733(11)	0.724(12)
raw X V_{HOM}	0.581(18)				
corr X V_{HOM}	0.605(19)				

5.3.2 Replicas of QD 3

In Fig. S9 we show a graph similar to Fig. 1 c) and d) from the main text but for QD 3. Recording and reconstruction of the graphs was explained above and in the main manuscript. Figure S9 a) shows spectra of every replica pair under TPE for a certain voltage, whereas b) shows the energy shift of each emission line as a function of the voltage. Compared to QD 1 of the main paper, the first replica is much more pronounced, while especially the third replica is only visible in a narrow voltage range. For $V = 0.90\,\mathrm{V}$ (orange) only replica 0 is visible. For $V = -1.60\,\mathrm{V}$ (green) and $V = -3.00\,\mathrm{V}$ (blue), three (replica 1-3) and one (replica 2, $10\times$ enhanced in brightness) replicas are visible. Figure S10 shows that the energy shifts of the replicas can also be explained by the field contribution from additional holes, as discussed above.

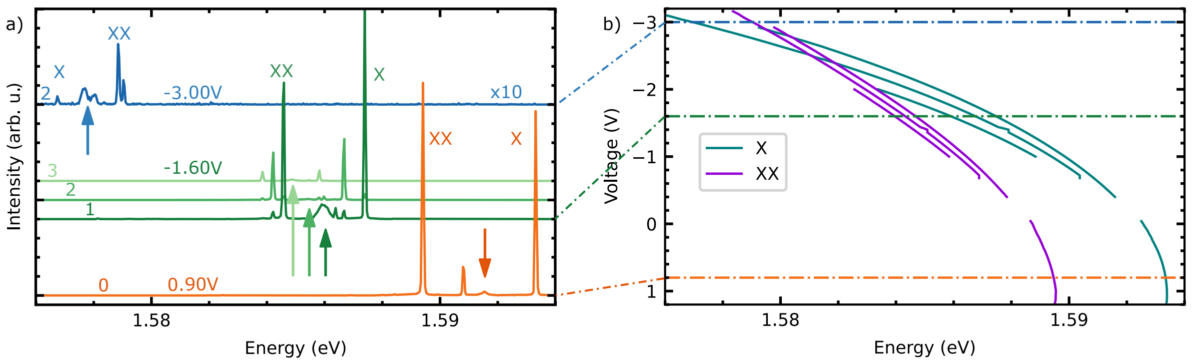

Figure S9: a) Spectra of QD 3 at three different voltages under π-pulse two-photon excitation. Depending on the voltage, several XX-X replicas (labeled 0–3) could be observed, sometimes simultaneously. For each spectrum, the laser energy was adjusted to match the two-photon resonance. Small contributions from other replicas come from phonon-assisted excitation. The wavelength of the excitation laser is indicated with arrows. b) fitted energies of the XX and X photons as a function of the applied voltage are shown. Each data point is extracted from a spectrum under two-photon excitation of the respective replica.

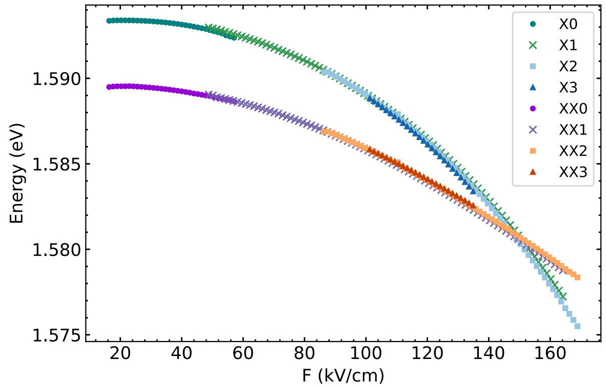

Figure S10: Emission energy as a function of the total electric field F for QD 3. The electric field contains the contribution of one, two or three positive charges located at a distance $\delta_0 + \delta$ from the QD center, where δ is the distance of the hole to the interface below the QD layer. The δ_0 is set to 11.5 nm, which is roughly the distance from the interface between the $Al_{0.15}Ga_{0.85}As$ and the $Al_{0.33}Ga_{0.67}As$ layers to the center of the QD.

References

(S1) Mooney, P. M. Deep donor levels (DX centers) in III-V semiconductors. *Journal of Applied Physics* **1990**, *67*, R1–R26.

(S2) Muñoz, E.; Calleja, E.; Izpura, I.; García, F.; Romero, A. L.; Sánchez-Rojas, J. L.; Powell, A. L.; Castagné, J. Techniques to minimize DX center deleterious effects in III-V device performance. *Journal of Applied Physics* **1993**, *73*, 4988–4997.

(S3) da Silva, S. F. C.; Undeutsch, G.; Lehner, B.; Manna, S.; Krieger, T. M.; Reindl, M.; Schimpf, C.; Trotta, R.; Rastelli, A. GaAs quantum dots grown by droplet etching epitaxy as quantum light sources. *Applied Physics Letters* **2021**, *119*.

(S4) Trellakis, A.; Zibold, T.; Andlauer, T.; Birner, S.; Smith, R. K.; Morschl, R.; Vogl, P. The 3D nanometer device project nextnano: Concepts, methods, results. *Journal of Computational Electronics* **2006**, *5*, 285–289.

(S5) Birner, S.; Zibold, T.; Andlauer, T.; Kubis, T.; Sabathil, M.; Trellakis, A.; Vogl, P. nextnano: General Purpose 3-D Simulations. *IEEE Transactions on Electron Devices* **2007**, *54*, 2137–2142.

(S6) Wolfe, C.; Korn, D.; Stillman, G. Silicon as a residual donor in high-purity GaAs. *Applied Physics Letters* **1974**, *24*, 78–80.

(S7) Ferreira da Silva, A.; Pepe, I.; Sernelius, B. E.; Persson, C.; Ahuja, R.; De Souza, J.; Suzuki, Y.; Yang, Y. Electrical resistivity of acceptor carbon in GaAs. *Journal of Applied Physics* **2004**, *95*, 2532–2535.

(S8) Hava, S.; Auslender, M. Velocity-field relation in GaAlAs versus alloy composition. *Journal of Applied Physics* **1993**, *73*, 7431–7434.

(S9) Brennan, K.; Hess, K. Theory of high-field transport of holes in Al0.45Ga0.55As. *Journal of Applied Physics* **1986**, *59*, 964–966.

(S10) Kodriano, Y.; Schwartz, I.; Poem, E.; Benny, Y.; Presman, R.; Truong, T. A.; Petroff, P. M.; Gershoni, D. Complete Control of a Matter Qubit Using a Single Picosecond Laser Pulse. *Physical Review B* **2012**, *85*, 1–5.

(S11) Muller, K.; Kaldewey, T.; Ripszam, R.; Wildmann, J. S.; Bechtold, A.; Bichler, M.; Koblmuller, G.; Abstreiter, G.; Finley, J. J. All Optical Quantum Control of a Spin-Quantum State and Ultrafast Transduction into an Electric Current. *Scientific Reports* **2013**, *3*, 1–7.

(S12) Santori, C.; Fattal, D.; Vučković, J.; Solomon, G. S.; Yamamoto, Y. Indistinguishable photons from a single-photon device. *Nature* **2002**, *419*, 594–597.

(S13) Zhai, L.; Nguyen, G. N.; Spinnler, C.; Ritzmann, J.; Löbl, M. C.; Wieck, A. D.; Ludwig, A.; Javadi, A.; Warburton, R. J. Quantum interference of identical photons from remote GaAs quantum dots. *Nature Nanotechnology* **2022**, *17*, 829–833.

(S14) Olivero, J. J.; Longbothum, R. L. Empirical fits to the Voigt line width: A brief review. *Journal of Quantitative Spectroscopy and Radiative Transfer* **1977**, *17*, 233–236.

(S15) Fry, P. W.; Itskevich, I. E.; Mowbray, D. J.; Skolnick, M. S.; Finley, J. J.; Barker, J. A.; O'Reilly, E. P.; Wilson, L. R.; Larkin, I. A.; Maksym, P. A.; Hopkinson, M.; Al-Khafaji, M.; David, J. P. R.; Cullis, A. G.; Hill, G.; Clark, J. C. Inverted Electron-Hole Alignment in InAs-GaAs Self-Assembled Quantum Dots. *Physical Review Letters* **2000**, *84*, 733–736.

(S16) Huang, H.; Csontosová, D.; Manna, S.; Huo, Y.; Trotta, R.; Rastelli, A.; Klenovský, P. Electric field induced tuning of electronic correlation in weakly confining quantum dots. *Physical Review B* **2021**, *104*, 165401.

(S17) Barker, J. A.; O'Reilly, E. P. Theoretical Analysis of Electron-Hole Alignment in InAs-GaAs Quantum Dots. *Physical Review B* **2000**, *61*, 13840–13851.

(S18) Jin, P.; Li, C. M.; Zhang, Z. Y.; Liu, F. Q.; Chen, Y. H.; Ye, X. L.; Xu, B.; Wang, Z. G. Quantum-Confined Stark Effect and Built-in Dipole Moment in Self-Assembled InAs/GaAs Quantum Dots. *Applied Physics Letters* **2004**, *85*, 2791–2793.

(S19) Finley, J. J.; Sabathil, M.; Vogl, P.; Abstreiter, G.; Oulton, R.; Tartakovskii, A. I.; Mowbray, D. J.; Skolnick, M. S.; Liew, S. L.; Cullis, A. G.; Hopkinson, M. Quantum-confined Stark shifts of charged exciton complexes in quantum dots. *Physical Review B* **2004**, *70*, 2–5.

(S20) Mar, J. D.; Baumberg, J. J.; Xu, X. L.; Irvine, A. C.; Williams, D. A. Precise Measurements of the Dipole Moment and Polarizability of the Neutral Exciton and Positive Trion in a Single Quantum Dot. *Physical Review B* **2017**, *95*, 201304.

(S21) Aberl, J.; Klenovský, P.; Wildmann, J. S.; Martín-Sánchez, J.; Fromherz, T.; Zallo, E.; Humlíček, J.; Rastelli, A.; Trotta, R. Inversion of the exciton built-in dipole moment in In(Ga)As quantum dots via nonlinear piezoelectric effect. *Physical Review B* **2017**, *96*, 045414.

(S22) Houel, J.; Kuhlmann, A. V.; Greuter, L.; Xue, F.; Poggio, M.; Gerardot, B. D.; Dalgarno, P. A.; Badolato, A.; Petroff, P. M.; Ludwig, A.; Reuter, D.; Wieck, A. D.; Warburton, R. J. Probing Single-Charge Fluctuations at a GaAs/AlAs Interface Using Laser Spectroscopy on a Nearby InGaAs Quantum Dot. *Physical Review Letters* **2012**, *108*, 107401.

(S23) Adachi, S. GaAs, AlAs, and Alx Ga1-x As: Material parameters for use in research and device applications. *Journal of Applied Physics* **1985**, *58*, R1–R29.

(S24) Strzalkowski, I.; Joshi, S.; Crowell, C. R. Dielectric Constant and Its Temperature Dependence for GaAs, CdTe, and ZnSe. *Applied Physics Letters* **1976**, *28*, 350–352.

(S25) Chuang, S. *Physics of Photonic Devices*; Wiley Series in Pure and Applied Optics; Wiley, 2009.

(S26) Kruck, T.; Babin, H. G.; Wieck, A. D.; Ludwig, A. Critical Aluminum Etch Material Amount for Local Droplet-Etched Nanohole-Based GaAs Quantum Dots. *Crystals* **2024**, *14*.
